# Development and Validation of Clinical Diagnostic Model for Girls with Central Precocious Puberty: Machine-learning Approaches

**DOI:** 10.1371/journal.pone.0261965

**Published:** 2022-01-21

**Authors:** Quynh Thi Vu Huynh, Nguyen Quoc Khanh Le, Shih-Yi Huang, Ban Tran Ho, Tru Huy Vu, Hong Thi Minh Pham, An Le Pham, Jia-Woei Hou, Ngan Thi Kim Nguyen, Yang Ching Chen

**Affiliations:** 1 Faculty of Medicine, Department of Pediatrics, University of Medicine and Pharmacy Ho Chi Minh City, Ho Chi Minh City, Vietnam; 2 Department of Nephrology and Endocrinology, Children’s Hospital 2, Ho Chi Minh City, Vietnam; 3 Professional Master Program in Artificial Intelligence in Medicine, College of Medicine, Medical University, Taipei, Taiwan; 4 Research Center for Artificial Intelligence in Medicine, Taipei Medical University, Taipei, Taiwan; 5 Translational Imaging Research Center, Taipei Medical University Hospital, Taipei, Taiwan; 6 School of Nutrition and Health Sciences, Taipei Medical University, Taipei, Taiwan; 7 Graduate Institute of Metabolism and Obesity Sciences, Taipei Medical University, Taipei, Taiwan; 8 Nutrition Research Center, Taipei Medical University Hospital, Taipei, Taiwan; 9 Faculty of Medicine, Department of Pediatric Surgery, University of Medicine and Pharmacy Ho Chi Minh City, Ho Chi Minh City, Vietnam; 10 Department of Pediatric Surgery, Children’s Hospital 2, Ho Chi Minh city, Vietnam; 11 Faculty of Medicine, Family Medicine Center, University of Medicine and Pharmacy Ho Chi Minh City, Ho Chi Minh City, Vietnam; 12 Department of Pediatrics, Cathay General Hospital, Taipei, Taiwan; 13 Department of Family Medicine, Taipei Medical University Hospital, Taipei, Taiwan; 14 Department of Family Medicine, College of Medicine, Taipei Medical University, Taipei, Taiwan; Florida International University, UNITED STATES

## Abstract

**Background:**

A brief gonadotropin-releasing hormone analogues (GnRHa) stimulation test which solely focused on LH 30-minute post-stimulation was considered to identify girls with central precocious puberty (CPP). However, it was tested using traditional statistical methods. With advanced computer science, we aimed to develop a machine learning-based diagnostic model that processed baseline CPP-related variables and a brief GnRHa stimulation test for CPP diagnosis.

**Methods:**

We recruited girls suspected of precocious puberty and underwent a GnRHa stimulation test at Children Hospital 2, Vietnam, and Cathay General Hospital, Taiwan. Clinical data, bone age measurement, and 30-min post-stimulation blood test were used to build up the predictive model. The candidate model was developed by different machine learning algorithms that were mainly evaluated by sensitivity, specificity, the area under the receiver operator characteristic curve (AUC), and F1-score in internal and external validation data to classify girls as CPP and non-CPP at different time-points (0-min, 30-min, 60-min, and 120-min post-stimulation).

**Results:**

Among the 614 girls diagnosed with PP, 524 (85.3%) had CPP. The random forest algorithm yielded the highest value of F1-score (0.976), specificity (0.893), positive predicted value (0.987), and relatively high value of AUC (0.972) that contributed to high probability to identify CPP. The performance metrics of the 30-min post-stimulation diagnostic model including sensitivity and specificity surpassed those of the 0-minute model (0-min) and were equivalent to those of the model obtained 60-min and 120-min post-stimulation. Hence, our machine learning-based model helps shorten the stimulation test to 30 minutes after GnRHa injection, in general, it requires 120 minutes for a completed GnRHa stimulation test.

**Conclusions:**

We developed a diagnostic model based on clinical features and a single sample 30-minute post-stimulation to identify CPP in girls that can reduce distress for children caused by multiple blood samplings.

## Introduction

Central precocious puberty (CPP) caused by the early activation of the hypothalamic-pituitary-gonadal axis is defined by the early development of secondary sex characteristics, acceleration of linear growth, advanced bone age, and a pubertal response to gonadotropin-releasing hormone (GnRH) test [1]. The annual incidence of CPP has substantially increased in children (mainly girls) [1]. CPP results in significantly shorter final height due to early closure of the epiphyses [2], which are calcified under the influence of estrogen [3,4]. In addition, children with early pubertal timing may be linked to psychosocial difficulties and negative health implications, including increased risk of type 2 diabetes [5], cardiovascular disease [5], depression [6], and premature death [7]. In girls, early puberty is associated with an increased risk of breast cancer [8], which urges a quick response in diagnosing and timely intervention.

In terms of CPP diagnosis, it is hard to distinguish actual CPP from precocious thelarche, which is often non-progressive or may resolve spontaneously. Hence, the GnRH stimulation test is needed as a gold standard to identify CPP [2]. Since in case of unavailable exogenous GnRH, the GnRH analogue (GnRHa) stimulation test can be substituted to diagnose CPP [3,9] and the cutoff peak LH level of >5 IU/L is widely used to identify CPP [3,10]. However, the stimulation test is constrained by different time points (at 30, 40, 60, and 120 min) and is expensive that carries a financial burden in those countries where health care service is not free. Intriguingly, the baseline luteinizing hormone (LH) level was a promising biomarker to diagnose CPP [11]. However, the diagnostic cutoff of baseline LH for CPP diagnosis varies from 0.1 to 1.5 IU/L, with a sensitivity ranging from 60% to 100% [10–15].

Regarding a brief GnRHa stimulation test, recent studies have raised the recommendation that a single sample 30- or 40-min post-stimulation is sufficient for CPP diagnosis in children [16–18] but using traditional statistical approach regardless of machine learning. Pan *et al*. [19,20] established a machine learning-based model using only baseline features, such as breast stages, pubic hair stages, body composition, basal serum LH, follicle-stimulating hormone (FSH), bone age assessment, pelvic ultrasonography to predict CPP in girls that did not entail the GnRHa stimulation test. No machine learning-based model conveying a single-sample 30-min post-stimulation in diagnosing CPP in girls has been established. Therefore, we aimed to develop and validate a diagnostic model for girls with CPP that handle multiple baseline CPP-related characteristics, and sex hormone measurements obtained 30 min post-stimulation using machine learning techniques.

## Materials and methods

### Participants and study design

This study was adhered to the guidelines of the Declaration of Helsinki and was approved by the Institutional Review Board of the University of Medicine and Pharmacy at Ho Chi Minh City, Vietnam, and Cathay General Hospital, Taiwan. Written consent has been obtained from each patient or their parents after fully explaining the purpose and nature of all procedures used.

**Fig 1:**
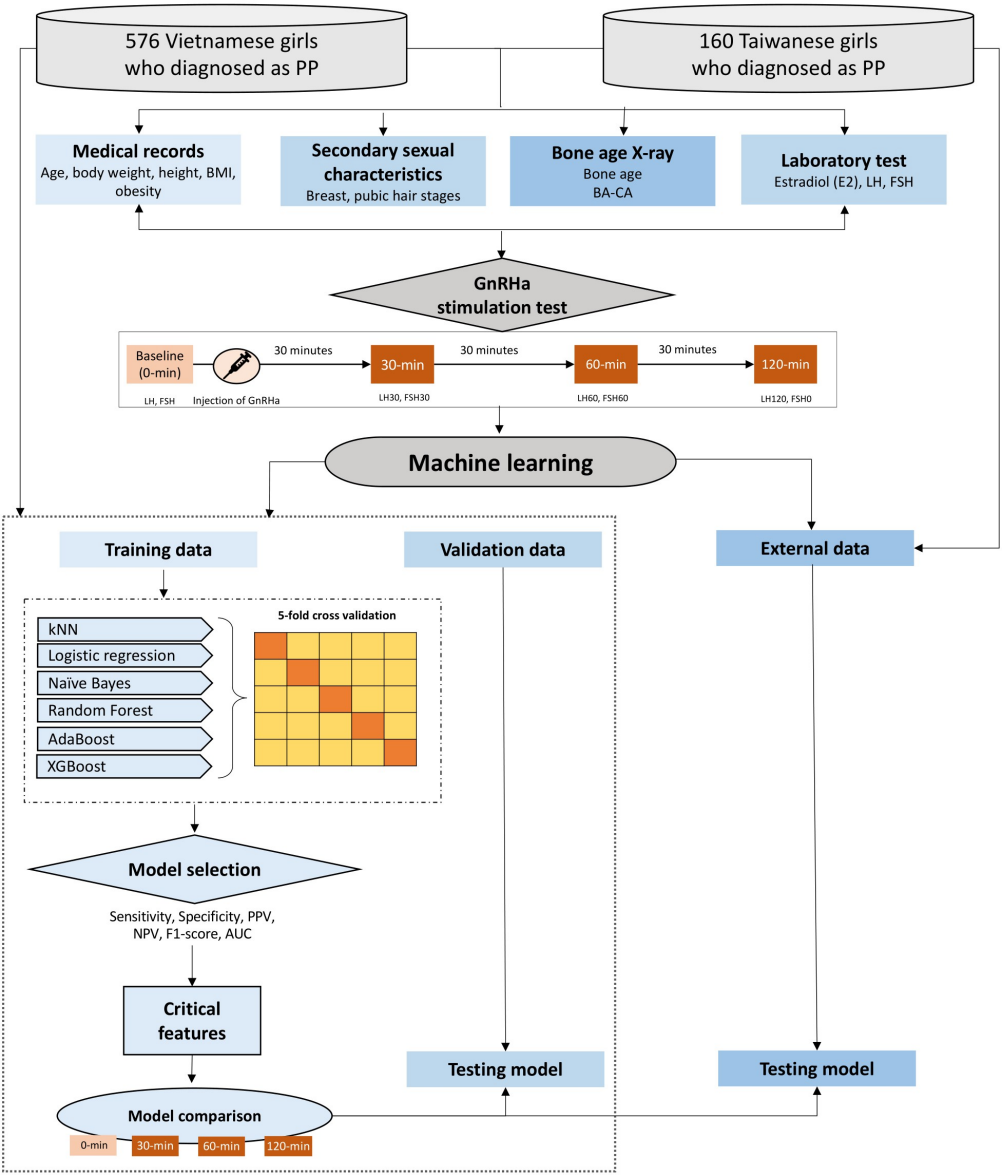
The flowchart of the study. *Definition of abbreviations*: AdaBoost, adaptive boosting; AUC, the area under the receiver operating characteristic curve; kNN, k-nearest neighbors; LIME, local interpretable model-agnostic explanations; XGBoost, extreme gradient boosting.

For this cross-sectional study, we recruited girls who had been diagnosed as having precocious puberty (PP) and were admitted to Children’s Hospital 2 in Southern Vietnam between January 2010 and December 2016 and Cathay General Hospital in Taipei, Taiwan, between March 2020 and February 2021. PP was defined when girls with at least one sign of puberty or who had progressive pubertal development associated with rapid growth in girls < 8 years or girls who began menarche < 9 years [2]. All girls with PP underwent the GnRHa stimulation test to identify CPP. We excluded girls diagnosed with congenital adrenal hyperplasia or hypothyroidism. Fig 1 illustrates the flowchart of the study (Fig 1).

### GnRH stimulation test and CPP diagnosis

The GnRHa stimulation test was performed in Children’s Hospital 2, Vietnam, and Cathay General Hospital, Taiwan. A standard dose of 0.1 mg of Triptorelin (Ipsen Pharma, Georges Gorse, Boulogne-Billancourt, France) was administered subcutaneously, with blood sampling conducted at the basal time point (0), 30, 60, and 120 min for LH measurement. All samples were analyzed LH and FSH using immunochemiluminometric assay (ICMA) (ARCHITECT i2000SR, 2016144 Abbott, Abbott Park, IL, USA). The ARCHITECT LH was designed to have an assay imprecision of ≤ 7% and ≤ 10% total coefficient variation (CV) for LH values ≤ 70 IU/L and LH values > 70 IU/L, respectively. The LH assay is designed to have a mean recovery of 100% ± 8% for LH levels across the range of 10–70 UI/L. Eventually, the detection value ranges from 0.09 IU/L to 250 IU/L. Meanwhile, the ARCHITECT FSH assay was standardized with the World Health Organization (WHO) First International Standard (IS) FSH 95/510, with the mean recovery of WHO 1st IS FSH being 96.05%. The operating range of assays was established by the precision profile, which was defined to have a total CV< 10%. ICMA FSH assay was compared to the AxSYM FSH assay, in which the serum detection ranges from 0.46 to 120.45 IU/L.

Girls received a CPP diagnosis if they exhibited the following criteria: (a) onset of secondary sexual characteristics including breast development and/or pubic hair development at age < 8 years and (b) peak LH level ≥ 5 IU/L combined with a ratio of peak LH to FSH value ≥ 0.6 after GnRHa stimulation test [2,21]. Girls who did not present with secondary sexual characteristics at age < 8 years and exhibited a negative response to the GnRHa stimulation test were considered non-CPP cases [3].

### Covariates

According to the Tanner stage, the presence of secondary sexual characteristics, such as breast development, pubic hair development, and menstruation/menarche, was recorded according to reports from parents and caregivers and examination by pediatric endocrinologists [22]. We measured the participants’ body weight and height to calculate the body mass index (BMI) [4]. Because in growing children BMI varies with age and sex, body weight, height, and BMI were converted into Z-scores according to the global application of the WHO Reference 2007 for children aged 5–19 years [23] that could help to compare weight status in different populations and to define obesity [24]. Obesity was defined as BMI ≥ +2 standard deviations (SDs, equivalent to BMI 30 kg/m^2^ at 19 years) from the BMI-for-age (BMI Z-score). Left-hand radiography was used to measure bone age according to the method proposed by Greulich and Pyle [25], and the difference between bone age and chronological age (BA-CA) was calculated as bone–age − chronological age (years). Laboratory measurements included basal serum estradiol, LH, and FSH before and after the GnRHa stimulation test.

### Model development based on machine learning algorithms: Training and validation

We excluded variables with missing values; accordingly, a robust model could be obtained without modification of the original variables. CPP-related variables [2] were finally selected for the development of the machine learning models. Specifically, nine variables were extracted from the participants’ clinical records: age (years), body weight (Z-score), height (Z-score), BMI (Z-score), obesity (yes/no), breast development (Tanner stages 1 to 5), pubic hair (Tanner stages 1 to 5), menstrual/menarche (yes/no), BA-CA (years). The remaining five variables were basal estradiol, LH, and FSH levels and LH and FSH levels 30 min after stimulation test.

In terms of different theories attributed by the accuracy, high speed, and simplicity, we evaluated the performance of the models by using different machine learning and ensemble learning algorithms, including k-nearest neighbors (kNN), logistic regression (LR), random forests (RF), adaptive boosting (AdaBoost), and extreme gradient boosting (XGBoost) algorithms [26–28].

Data of 576 Vietnamese participants were split into 75% for the training dataset and 25% for testing model performance (internal validation). Besides, we tested the candidate model by using 38 Taiwanese participants as external validation. Each data set comprised a case/control design corresponding to CPP and non-CPP cases. Cross-validation was used to assess how the proposed system results will generalize the CPP and non-CPP subjects. All the samples were randomly allocated to five subsets with an equal number of samples during this process. Then, we trained five separate recognition systems using four out of the five subsets and validated the fifth holdout subset.

Due to the model referenced to the GnRHa stimulation test, sensitivity, and specificity refer to the proportion of those who were diagnosed as CPP (true positive rate) and non-CPP (true negative rate), respectively were considered. The accuracy is commonly used to measure the performance of the binary classification model. However, as our dataset is imbalanced in two classes (CPP and non-CPP) and there is a trade-off between increasing the sensitivity and the precision (or predicted positive value, PPV), we consider F1-score as a fairer metric to select the candidate model than the accuracy due to F1-score is the weighted average of precision and recall (or sensitivity) that takes both false positives and false negatives into account. These evaluation measurements are defined as follows:

$$Sensitivity=\frac{TP}{TP+FN}$$

$$Specificity=\frac{TN}{TN+FP}$$

$$Positive\;predicted\;value\;(PPV)=\frac{TP}{TP+FN}$$

$$Negative\;predicted\;value\;(NPV)=\frac{TN}{TN+FN}$$

$$Accuracy\;(ACC)=\frac{TP+TN}{TN+TN+FP+FN}$$

$$F1-score=2*\frac{Sensitivity*Precision}{Sensitivity+Precision}$$

where TP, TN, FP, and FN denote true positives, true negatives, false positives, and false negatives. Moreover, to overcome the possibilities of the imbalance dataset, we reported the receiver operating characteristic (ROC) curve and the area under the ROC curve (AUC) values to see the overall performance at different threshold points.

In general, the standard GnRH stimulation test requires different time points (0 min, 30 min, 60 min, and 120 min) to identify CPP. To emphasize the efficacy of the candidate model which included baseline characteristics and FSH-, LH 30-min post-stimulation, the model performance was compared to those of the 60-min post-stimulation model that were added LH and FSH levels obtained 60 min after stimulation. Similarly, its model performance was compared to those of models deriving from FSH-, LH 120-min post-stimulation. All developed models were tested and applied on the internal validation (Vietnamese girls) and external validation (Taiwanese girls).

### Model interpretation

To overcome the disadvantages of the black-box machine learning model and provide physicians with more information on the prediction model in clinical practice, we used the local interpretable model-agnostic explanations (LIME) algorithm to interpret feature contributions for each prediction [19,29,30]. LIME analysis revealed the probability score as well as the cutoff for prediction by the model. After installing the LIME package, 50% of the data was used for testing and visualization in our model interpretation. LIME analysis was conducted on both testing and validation data to examine the different effects of the samples.

### Statistical analysis

Data for continuous variables and categorical variables are presented as the mean ± SD and percentages. Pearson’s χ2 test was used to analyze categorical variables, and the independent Student’s t-test analyzes continuous variables. All computations and visualizations were analyzed using Python with packages such as Scikit-learn, Pandas, Lime, and Matplotlib.

## Results

### Study characteristics

Out of 614 girls with PP (mean age 7.4 ± 1.7 years), 85.3% were diagnosed as having CPP. Table 1 presents the numbers of girls with and without CPP in the discovery and validation data sets. As shown in Table 1, 25 variables significantly differed between girls with and without CPP; exceptions were the Z-scores of weights and BMI and the baseline LH-to-FSH ratio (Table 1). Besides, girls in the external validation dataset were older than girls in the internal validation dataset (8.4 ± 1.1 vs. 7.5 ± 1.4, *p*<0.001). Also, there were significant mean differences in BA-CA, body weight, height, basal estradiol, FSH 60 min, FSH 120 min, and LH 120 min between girls in the external validation dataset and those in the internal validation dataset (data not shown).

**Table 1:** Basic characteristics of girls who underwent the GnRHa stimulation test in discovery and testing data set.

Variables	Discovery data set		Testing data set				*P* value^a^
	Vietnamese patients (n = 432)		Vietnamese girls (n = 144)		Taiwanese girls (n = 160)		
	CPP (n = 376)	Non-CPP (n = 56)	CPP (n = 122)	Non-CPP (n = 22)	CPP (n = 95)	Non-CPP (n = 65)	
Medical record							
Mother AAM (y)	13.8±1.4	14.6±1.3	13.9±1.2	14.1±2.4	11.6±1.0	12.1±1.9	<0.01
Age (y)	7.2±1.8	7.5±1.5	7.4±1.4	7.6±1.0	8.5±1.1	8.4±1.2	<0.01
Age < 2 y, n (%)	5,1.3%	7,58.4%	1,0.8%	3,13.6%	0	0	<0.01
Age 2–6 y, n (%)	24,6.4%	23,41.1%	10,8.2%	6,27.2%	3 (75.0%)	1 (25.0%)	
Age > 6 y, n (%)	347,52.4%	26,46.4%	111,91.0%	13,59.2%	92 (59.0%)	64 (41.0%)	
Weight (kg)	31.7±6.8	22.2±8.2	31.4±7.2	24.±10.	35.6±8.6	35.0±7.1	<0.01
Weight (Z-score)	1.3±1.0	1.1±0.9	1.3±1.0	1.2±0.9	0.9±1.1	0.8±1.5	0.20
Height (cm)	131.3±12.5	113.6±17.3	131.1±9.6	114.2±20.6	138.5±8.2	136.2±6.5	<0.01
Height (Z-score)	1.2±1.1	0.8±0.9	1.2±1.1	1.1±1.0	0.7±1.1	0.4±1.1	0.02
BMI (kg/m^2^)	17.9±2.4	16.5±2.7	18.1±2.7	17.2±3.1	18.3±2.8	18.8±3.3	0.03
BMI (Z-score)	0.9±1.1	0.9±1.0	0.8±1.3	0.8±0.9	0.7±1.1	0.7±1.9	0.90
Obesity (yes), n (%)	197,91.6%	18,8.4%	66,45.8%	6,27.3%	12 (13.4%)	77 (86.6%)	<0.01
Breast development (Stage 2, 3, 4)	375,99.7%	46,82.1%	122100%	16,72.7%	64 (57.1%)	48 (42.8%)	<0.01
Pubic hair (Stage 2, 3, 4)	127,33.8%	6,10.7%	35,28.6%	5,22.7%	15 (83.3%)	3 (16.7%)	<0.01
Menarche (yes), n (%)	45,11.9%	16,28.6%	11,9.0%	4,18.2%	33 (75.0%)	11 (25.0%)	<0.01
Bone age X-ray image							
Bone age (y)	9.9±1.8	5.7±2.4	9.8±1.7	5.8±2.8	9.3±1.5	8.7±1.1	<0.01
BA-CA (y)	2.3±1.1	0.3±1.1	2.3±1.1	0.1±0.9	1.6±0.9	1.8±0.7	<0.01
Laboratory test							
E2 (pg/mL)	39.9±23.7	54.0±44.6	0.7±0.4	0.5±0.5	28.9±17.5	22.8±15.5	0.03
Basal FSH (IU/L)	3.5±1.7	1.6±1.5	3.5±1.8	1.6±1.4	3.7±3.5	2.9±2.1	<0.01
Basal LH (IU/L)	1.7±2.7	0.1±0.1	1.4±1.6	0.1±0.4	3.0±5.0	2.2±1.8	<0.01
Basal LH/FSH	0.5±1.5	0.7±3.3	0.5±1.9	2.1±9.4	0.9±0.8	1.1±1.1	0.39
GnRHa stimulation test							
FSH 30 min (IU/L)	9.7±4.7	6.0±5.1	9.2±4.6	6.6±4.8	18.1±59.8	11.5±4.5	<0.01
LH 30 min (IU/L)	21.3±19.1	1.9±1.6	19.8±17.8	2.0±1.7	22.9±26.8	17.8±17.7	<0.01
LH/FSH 30 min	2.2±2.6	0.4±0.3	2.0±1.5	0.3±0.2	1.9±2.0	1.5±1.3	<0.01
FSH 60 min (IU/L)	12.6±6.4	8.0±6.7	11.5±5.8	8.8±6.5	13.0±4.7	12.9±4.5	<0.01
LH 60 min (IU/L)	24.4±21.5	2.5±2.3	21.7±19.2	2.4±2.0	19.8±24.9	14.5±24.8	<0.01
LH/FSH 60 min	2.0±2.8	0.4±0.7	1.8±1.3	0.3±0.1	1.5±1.7	1.2±1.0	<0.01
FSH 120 min (IU/L)	14.8±6.9	10.±9.2	13.4±6.2	12.±9.1	9.1±3.2	9.4±2.1	<0.01
LH 120 min (IU/L)	21.9±18.9	2.6±2.7	19.7±18.2	2.5±2.1	10.9±3.1	10.5±2.5	<0.01
LH/FSH 120 min	1.5±1.1	0.4±0.8	1.4±1.1	0.2±0.1	1.2±1.3	1.2±0.4	<0.01

*Abbreviation definition*: AAM, age at menarche; BA-CA, difference between the bone age and the chronological age; BMI, body mass index; FSH, follicle-stimulating hormone; LH, luteinizing hormone; GnRHa, gonadotropin-releasing hormone analogue.

^a^ Differences in baseline characteristics between girls with and without CPP in the entire population (593 girls with CPP and 143 girls without CPP).

### Assessment of different machine learning-based diagnostic models of CPP in girls

**Fig 2:**
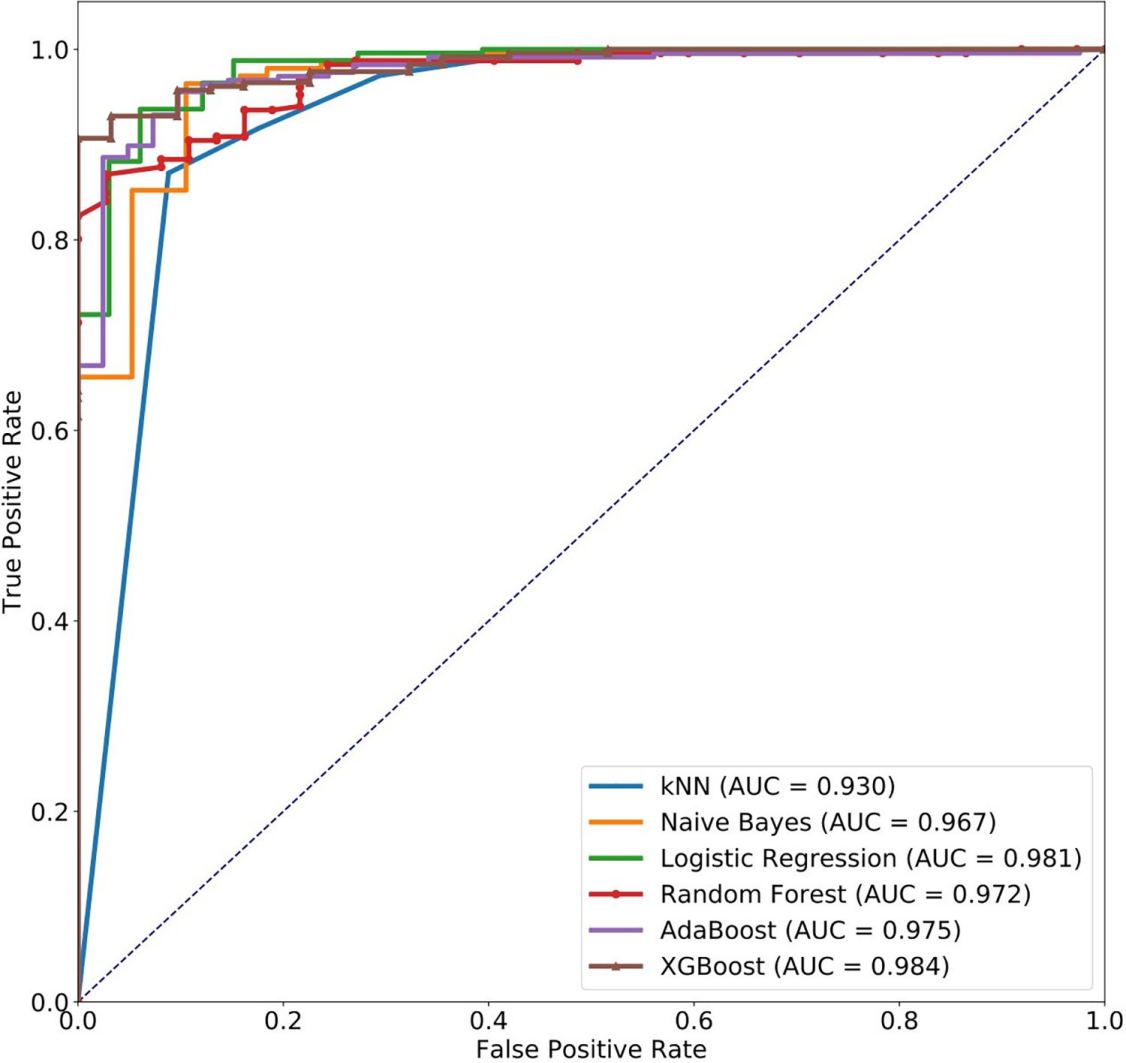
ROC curves of different algorithms in the training dataset. Predictive model included baseline characteristics and blood sampling 30 mins post-stimulation in the training dataset. *Definition of abbreviations*: AUC, an area under the receiver operating characteristic curve (ROC); kNN, k-nearest neighbors; AdaBoost, adaptive boosting; XGBoost, extreme gradient boosting.

Random Forest yielded the best result of all the algorithms in F1-score, specificity, and PPV (Table 2). The Random Forest also exhibited high sensitivity (0.961) and a high AUC value (0.984) (Fig 2).

**Table 2:** Model performance of different algorithms in the training dataset (n = 432).

Different algorithms mean (SD)	Sen.(%)	Spec. (%)	PPV (%)	NPV (%)	F1-score
**14-variable model** ^**a**^					
kNN	94.5±1.0	66.8±8.5	95.2±1.8	87.9±1.4	0.948±0.009
Naïve Bayes	98.0±1.6	55.9±6.4	89.4±3.9	90.8±1.1	0.934±0.019
Logistic Regression	96.9±2.0	81.4±6.4	97.3±0.9	89.8±1.6	0.971±0.011
Random Forest	96.6±1.5	89.3±7.9	98.7±0.9	76.7±10.6	0.976±0.012
AdaBoost	97.6±0.6	81.1±2.4	97.1±0.6	90.6±0.5	0.973±0.005
XGBoost	97.4±1.8	79.9±12.4	96.5±2.6	90.2±1.5	0.969±0.011
**Three-variable model** ^**b**^					
kNN	97.6±1.4	84.4±5.2	97.6±1.1	83.6±10.0	0.976±0.006
Naïve Bayes	97.8±2.1	74.0±11.4	95.5±2.0	85.5±13.8	0.966±0.018
Logistic Regression	97.1±1.9	86.0±6.6	98.1±0.7	80.0±13.5	0.976±0.013
Random Forest	97.1±1.9	86.7±8.8	98.1±1.2	80.0±13.5	0.976±0.012
AdaBoost	97.4±1.3	84.0±5.5	97.6±1.1	82.0±9.3	0.975±0.007
XGBoost	97.6±1.1	82.7±5.5	97.3±0.9	83.8±7.8	0.975±0.006

^a^ Predictive model includes ages (yrs), BMI (Z-score), height (Z-score), body weight (Z-score), obesity (yes), breast stage (1 to 5), pubic hair stage (1 to 5), menarche (yes), BA-CA (yrs), estradiol (pg/mL), FSH (IU/L), LH (IU/L) at initial visit, and LH (IU/L) and FSH (IU/L) at 30^th^ min post-stimulation in the training dataset.

^b^ Predictive model includes BA-CA (yrs), estradiol (pg/mL), LH (IU/L) at initial visit, and LH (IU/L) at 30^th^ min post-stimulation in the training dataset.

*Definition of abbreviations*: Sen., sensitivity; Spe., specificity; PPV, positive predictive value or precision; NP, negative predictive value.

### Optimal features for predicting CPP in girls: Training and validation

**Fig 3:**
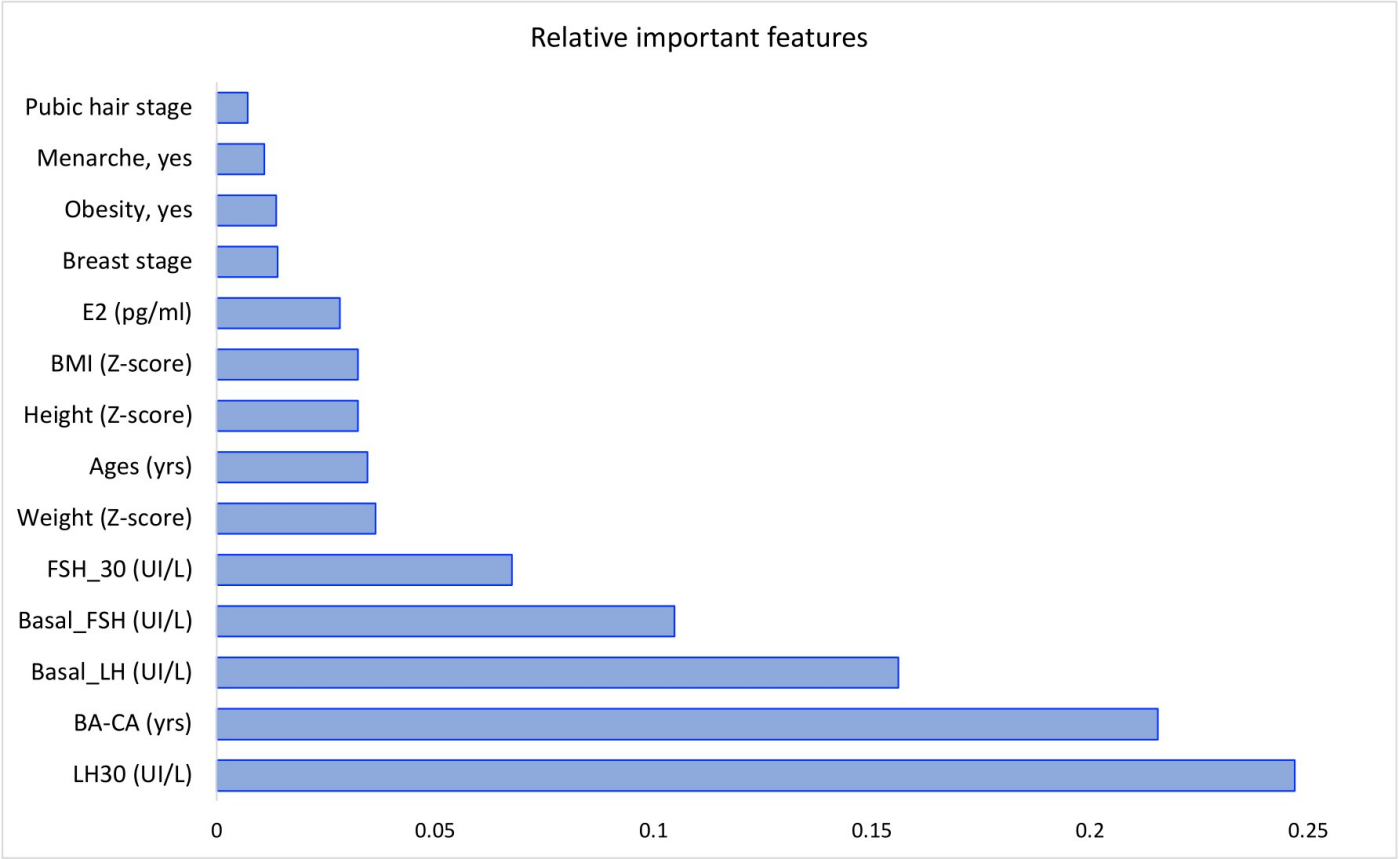
Feature importance ranking. This figure lists the relative importance of baseline characteristics and 30-minute post-stimulated gonadotropin levels in the developed machine learning-based model for the CPP diagnosis in girls. *Definition of abbreviations*: BA-CA, the difference between the bone age and the chronological age; BMI, body mass index; E2, basal estradiol; FSH, follicle-stimulating hormone; LH, luteinizing hormone.

We evaluated the ability of the correlation algorithm to find an optimal set of features. To assist physicians in the clinic when timely detection is necessary, we focused on the critical variables, including 30-min post-stimulation LH ("LH30"), "BA-CA" and “Basal_LH” that were the three top-ranking variables associated with accurate prediction in our model (Fig 3). The models that only included these critical features improved the sensitivity (from 0.966 ± 0.015 to 0.971 ± 0.019) and NPV (from 0.767 ± 0.106 to 0.800 ± 0.135) that helps a higher chance to support negative prediction (Table 2).

**Fig 4:**
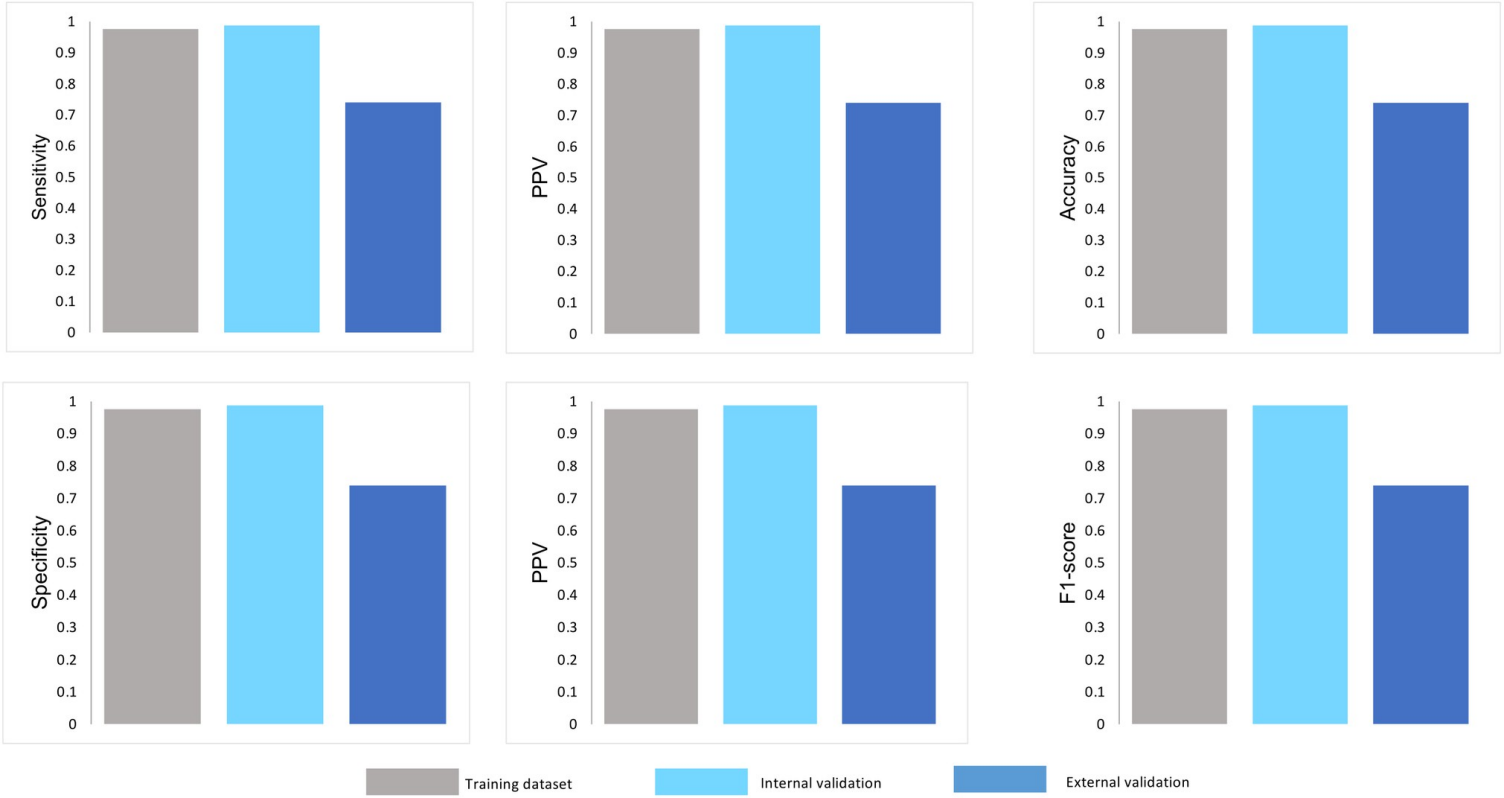
Comparison of the performance of the three-variable proposed model in a different dataset. *Definition of abbreviations*: NPV, negative predictive value; PPV, positive predictive value.

Fig 4 compares the model performance of three critical variables in different datasets (training, internal validation, and external validation). Most metrics of our proposed model were comparable between training and internal validation in terms of sensitivity, specificity, PPV, F1-score. Meanwhile, the model tested in the external population showed a lower specificity, PPV, accuracy, and F1-score, except for NPV at the highest value (100%) which returns no false-negative (Fig 4).

### Comparison of different time points used in the models for CPP in girls

**Fig 5:**
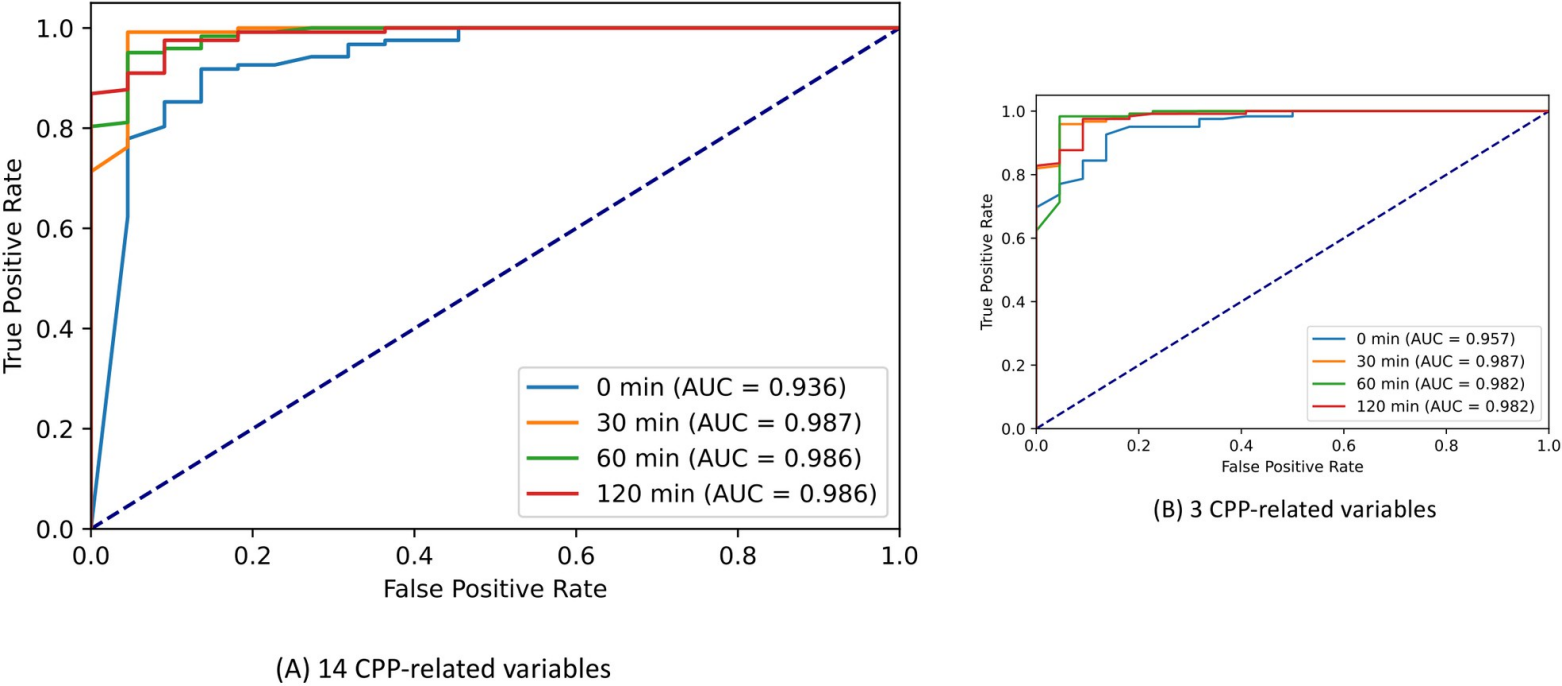
AUC values of predictive models in a different time point in the training dataset. Model performance of the 14-variable proposed model (A) and three-variable model (B) at 0 min, 30^th^ min, 60^th^ min, and 120^th^ min post-stimulation in the training dataset. *Definition of abbreviation*: AUC, an area under the receiver operating characteristic curve.

We built different models in different time points, including baseline (0-min), 30-, 60-, and 120-min post-stimulation. Fig 5 shows that the AUC value of the 30-min diagnostic model was higher than those of 0-min, 60-min, and 120-min post-stimulation models at both 14-variable and three-variable models (Fig 5). Then we validated the diagnostic model conveying three critical variables in different time points.

**Fig 6:**
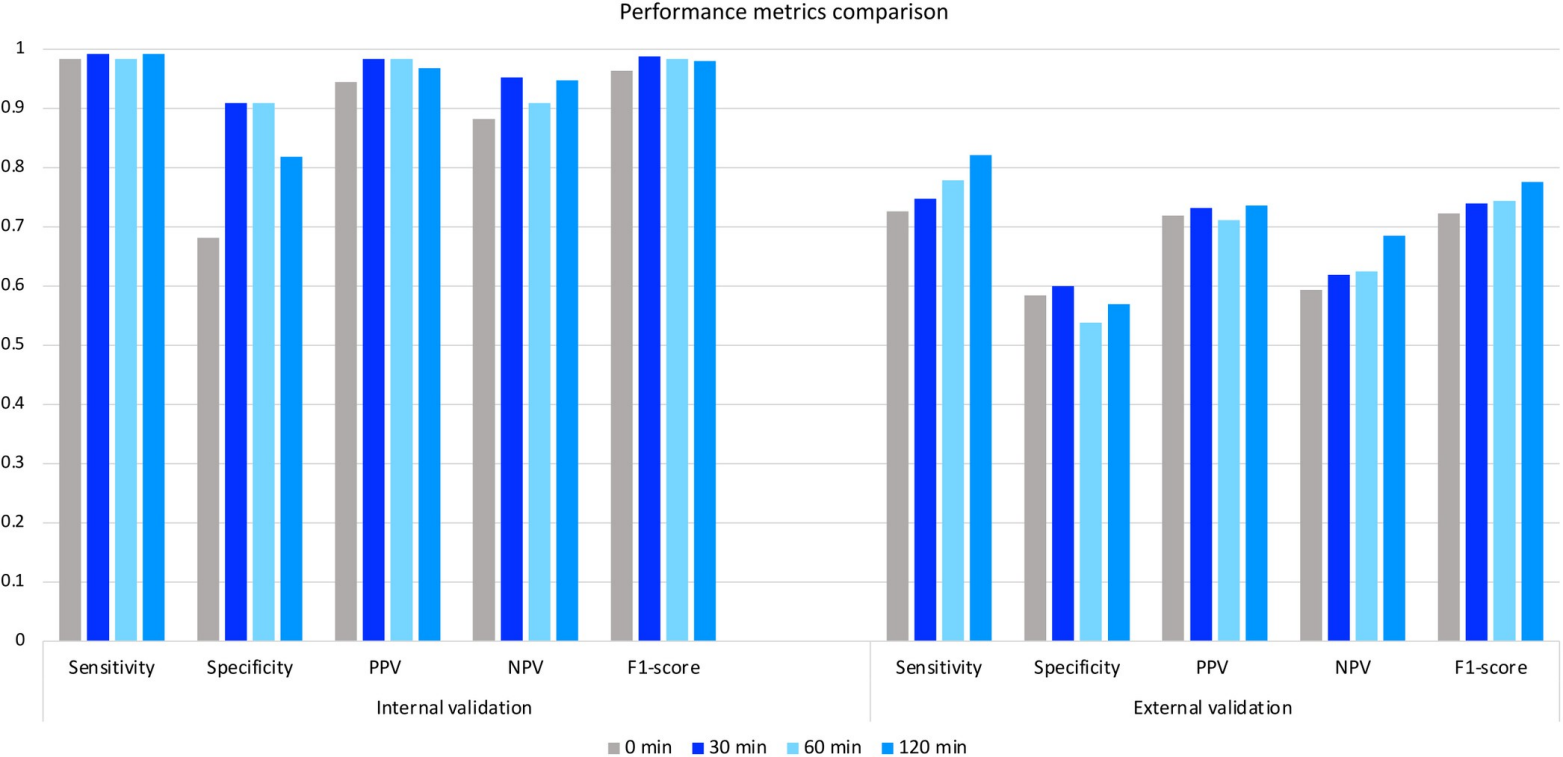
Performance metrics of and three-variable predictive model in the internal and external dataset. Model performance of the three-variable model was evaluated at 0 min, 30^th^ min, 60^th^ min, and 120^th^ min post-stimulation in the internal and external dataset. *Definition of abbreviations*: NPV, negative predictive value; PPV, positive predictive value.

The internal validation results show no differences in sensitivity, PPV, NPV, and F1-score between 30-min and 60-min and 120-min models. Compared to the baseline (0-min) model, the 30-min model improved the specificity (0.682 vs. 0.910), which is necessary for a diagnostic test to identify true negatives (girls without CPP). However, its specificity was equal to those of 60-min and 120-min models (Fig 6). Meanwhile, most performance metrics resulting from external validation were lower than those of internal validation. At last, the 30-min model surpassed the baseline (0-min) model in all the performance metrics, especially specificity (60%) in which accurately identified 60% of girls with non-CPP as GnRHa-stimulation test negative. Compared to 60-min and 120-min models, the 30-min model performed equivalent values of PPV and F1-score (Fig 6). Herein, a simplified model at 30-min post-stimulation was sufficient to identify CPP in girls.

### Local interpretable model-agnostic explanations for interpretation

**Fig 7:**
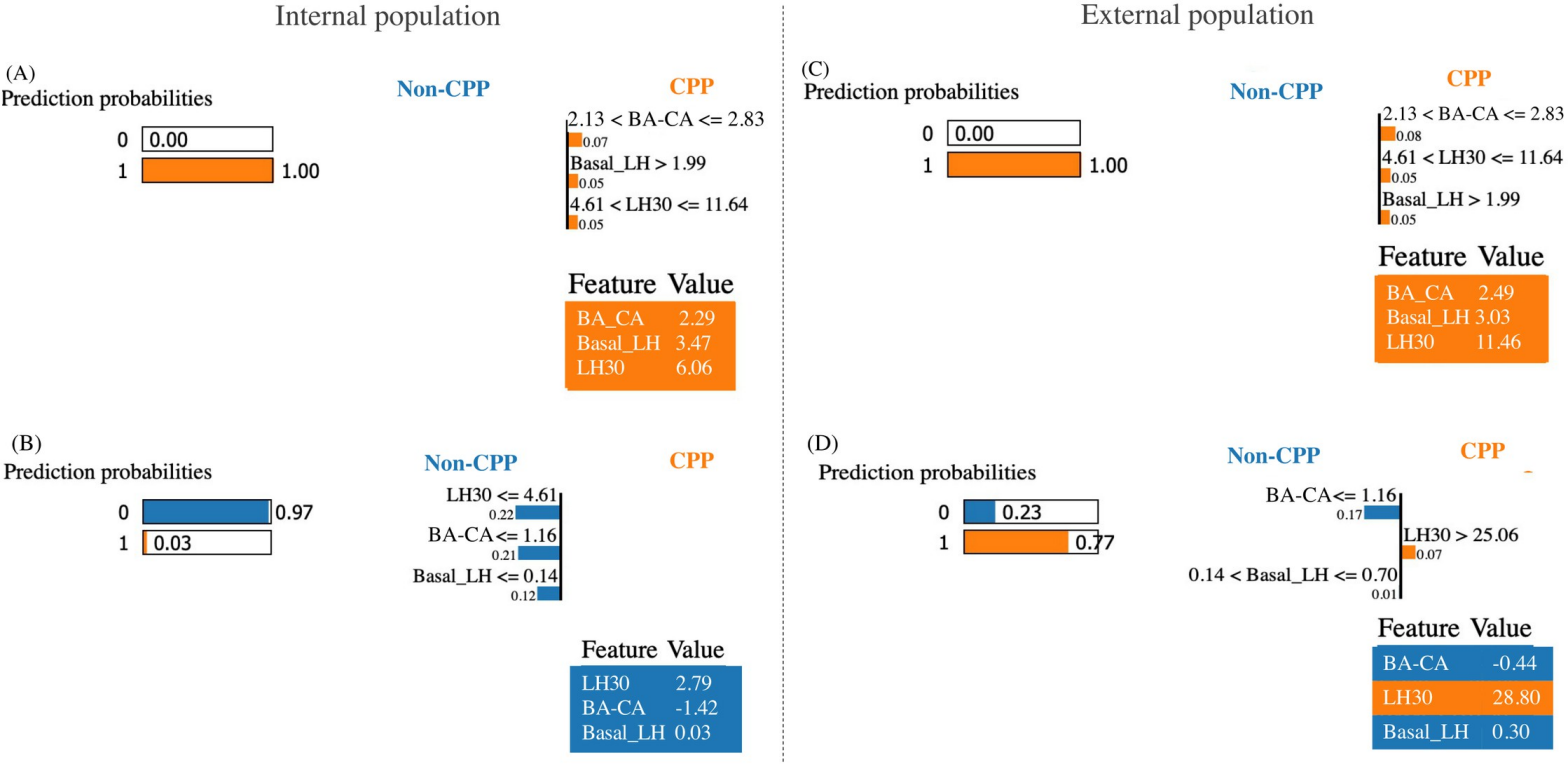
LIME analysis in different populations. LIME analysis for Vietnamese girls (A, B) and Taiwanese girls (C, D) according to positive and negative responses. *Definition of abbreviations*: BA-CA, the difference between the bone age and the chronological age; Basal_LH, luteinizing hormone level at baseline; CPP, central precocious puberty; LH30, luteinizing hormone level 30 min after stimulation.

Fig 7 illustrates one positive sample and one negative sample for both Vietnamese and Taiwanese girls. For positive prediction, “LH30” > 4.61 IU/L, “BA-CA” > 2.12 years, “Basal_LH” >1.99 IU/L supports to identify CPP with a probability of 100% (Fig 7A & 7C). For negative prediction, “LH 30” < 4.61 IU/L, “BA-CA” < 1.16 years, and “Basal_LH” ≤ 0.14 IU/L supports a non-CPP diagnosis with a probability of 97%. Considerably, “LH30” > 25.06 IU/L supports positive prediction with a probability of 77% that emphasizes the important role of “LH30” in the candidate model (Fig 7D).

## Discussion

Girls suspected CPP requires a time-consuming GnRHa stimulation test, which is a gold standard for CPP diagnosis. This study developed a machine learning-based model employing different clinical data sources, especially a brief GnRHa stimulation test (only 30-min post-stimulation) to determine girls with CPP. The model was achieved according to the highest performance metric of F1-score (0.976) and a high AUC value (0.972). A significant strength of our study is conveying a simple GnRHa stimulation test to build up an appropriate model for CPP diagnosis. The model was tested in the internal and external data. Notably, the performance metrics of the candidate model surpassed those of the baseline (0-min) model and were equivalent to those of 60-min and 120-min models. Our finding indicates the trend of CPP diagnosis using a machine learning-based model that helps make a better diagnosis than traditional methods.

As stated, the LH obtained after 30- or 40-min post-stimulation was recommended to diagnose CPP with an extensive range of sensitivity and specificity (90%–99% and 81%–100%, respectively [2,16,17,31,32] regardless of the machine learning application. Pan *et al*. [19,20] and Wenyong et al [33] firstly applied machine learning to propose a prescreening tool for CPP in girls, but they solely determined baseline clinical characteristics without taking the GnRHa stimulation test. To strengthen it, added FSH- and LH 30-min post-stimulation can be considered in developing a diagnostic model of CPP in girls. The current model yielded a higher AUC value ranging from 0.981 to 0.984 than those of the model proposed by Pan *et al*. [19,20], especially in terms of sensitivity and specificity, which are crucial metrics indicating the number of correctly predicted CPP cases [34]. These differences were possibly derived from a single sampling 30-min post-stimulation which is the gold standard in clinical practice for CPP diagnosis in the present study. Compared to previous studies, the similarity of machine learning algorithm was used, for instance, XGBoost [19,20], Random Forest [19], logistic regression [33]. Notably, apart from given CPP-related variables consisting of sexual characteristics (breast stages, pubic hair stages), sexual hormones, gonadotropins, and bone-age assessment at baseline in most studies [19,20,33], pelvic ultrasonography [20,33] was not assessed in the current study. Indeed, Pan et al [20] declared that pelvic ultrasonography alone could not differentiate girls with CPP from non-CPP well. Therefore, our 14-variable model was the best option in distinguishing between positive and negative responses to the stimulation test with the F1-score of 0.976 and AUC of 0.972 based on the Random Forest algorithm, in line with the previous finding [19].

Regarding the importance ranking of the top three features, the performance metrics of the three-variable model, including basal LH, BA-CA, and LH 30-min post-stimulation were slightly lower than those of a 14-variable model. The three-variable model was doubly validated in internal data and external data set. The specificity and NPV of the model derived from internal validation data increased up to 0.910 and 0.952, respectively, which helps to indicate the accuracy of the CPP diagnostic model in terms of "no false negative." However, the model performance deriving from external data showed a lower specificity, PPV, and F1-score. These differences may be attributed to significant age differences between two populations in the validated data set that led to differences in average values of the following important features, such as BA-CA, basal LH, basal FSH, FSH_30. Additionally, different race/ethnicity factors could affect pubertal development. Last but not least, the performance metrics of our 30-min model were much higher than those of the baseline (0-min) model and were equivalent to the 60-min and 120-min models in terms of AUC (Fig 5), sensitivity, specificity, PPV, NPV, and F1-score (Fig 6). Herein, our diagnostic model combining basal LH, BA-CA, and LH 30-min post-stimulation is reliable to identify CPP in girls.

LH level obtained 30 minutes after stimulation was crucial in identifying girls with CPP [17,18]. The cutoff value of > 5 IU/L for the peak LH level of stimulation test is commonly used to confirm CPP [3,10] in the clinic. After the GnRHa stimulation test, the peak LH level was observed at 30 min post-stimulation [14] that raised the significant diagnostic value of 30-min blood sampling. Therefore, our model interpreted the contributions of features using the reference of peak LH level (5 IU/L) [3,10]. As stated, the “LH30” > 4.61 IU/L (~5 IU/L) positively contributed to the high predicted probability of diagnosing CPP in girls. By contrast, “LH30” < 4.61 IU/L contributed a 22% probability to predict non-CPP. Secondly, “BA-CA” was the second-top rank feature. Although premature adrenarche is often associated with BA-CA ≥ 2 years [35], a recent study reported that BA-CA was the most effective predictor of positive response to the GnRHa stimulation test [36]. We found that girls with CPP presented with greater BA-CA than those without CPP, in line with previous findings [36,37]. LIME analysis revealed that girls with a “BA-CA” of > 2.13 years were at high risk for CPP in both populations, whereas a “BA-CA” of < 1.16 years helped exclude CPP diagnosis. In line with a previous study, BA-CA could be an additional factor in conjunction with the 30-min post-stimulation LH to diagnose CPP in girls [38]. Also, another feature is basal LH which has been served as a biomarker for CPP diagnosis and the diagnostic cutoff of basal LH varies from 0.1 to 1.5 IU/L (9). LIME revealed that the “basal LH” >1.99 IU/L contributed a 5% probability of identifying CPP, and the “basal LH” < 0.14 IU/L supported the negative prediction. Fig 7 also illustrates that the different combinations of variables may produce different prediction probabilities with similar predictive accuracy. Notably, out of secondary sexual characteristics needed, our candidate model conveying “basal LH”, “BA-CA”, and “LH30” that are clinically important features demonstrated that our model is reliable and effective in diagnosing CPP in girls.

The present study had several limitations. First, owing to this being a cross-sectionally study, we did not include the growth velocity rate which was modulated by the early activation and maturation of the hypothalamic-pituitary-gonadal axis [3]. Therefore, linear growth acceleration could be used in further study. Also, we could not differentiate progressive from non-progressive PP to avoid unnecessary treatment for the latter in the current study. Secondly, the performance metrics of our model derived from the external data were lower than expected. It was possibly due to the smaller sample size. Though the number of CPP cases has substantially increased [1], the overall incidence was quite low (15.3 per 100,000 girls) in Asia [39] that made it difficult to have a larger sample size in Taiwan that requires a further study to investigate the annual incidence rate of CPP in this country. In addition, the high prevalence of CPP in this population (68.4%, 26/38), at the expense of a negative association between disease prevalence and diagnostic specificity [40]. Another reason may result from race/ethnicity factor that affects pubertal development [41]. However, the specificity, PPV, NPV, and F1-score of models set at the 30-min post-stimulation was higher than those at baseline (0-min). Thirdly, we recruited only girls in a single-center, one of the most prominent pediatric hospitals in Southern Vietnam. Hence, the current diagnostic model may not be well-performed in other areas or countries, which merits further study in a larger population and multi-country.

In conclusion, the present study is the first to develop a machine learning-based diagnostic model consisting of different data sources, especially a brief GnRHa stimulation, thereby it helps to reduce the time-consuming and distress caused by the GnRHa stimulation test for children. Notably, our diagnostic model conveyed the important clinic features, such as basal LH, BA-CA, and 30-min LH, that make it reliable and effective in diagnosing CPP in girls.
